# Awareness, Knowledge, and Attitude Towards Urinary Tract Infections: An Appraisal From Saudi Arabia

**DOI:** 10.7759/cureus.49352

**Published:** 2023-11-24

**Authors:** Abdullatif K Almaghlouth, Reda A Alkhalaf, Abdulaziz A Alshamrani, Jumanah A Alibrahim, Baker S Alhulibi, Ali Y Al-Yousef, Aisha K Alamer, Saud M Alsuabie, Sukainah M Almuhanna, Abdullah D Alshehri

**Affiliations:** 1 Urology, King Faisal University, Al-Ahsa, SAU; 2 Internal Medicine, King Faisal University, Al-Ahsa, SAU

**Keywords:** general public awareness, awareness, bacterial urinary tract infection, infection, urinary tract infection, uti, uti: urinary tract infection

## Abstract

Introduction

Urinary tract infections (UTIs) are a common global health issue, yet awareness and knowledge about UTIs among the general population can vary widely. This study aimed to assess the awareness, knowledge, and attitudes regarding UTIs among Saudi Arabian citizens residing in Al-Ahsa, Saudi Arabia.

Methods

A descriptive cross-sectional study was conducted among Saudi Arabian citizens aged 18 and above residing in Alhassa. A structured questionnaire was used to collect data on participants' awareness, knowledge, attitudes, and experiences related to UTIs. Data were analyzed using IBM® SPSS® Statistics.

Results

The study included 445 participants, predominantly males, with 279 (62.7%) and a range of educational backgrounds. Approximately 302 (70.1%) of the participants were aware of UTIs. However, misconceptions about the definition of UTI and its risk factors were common. Most participants recognized bacteria as the primary cause of UTIs, with 261 (58.7%) identifying this factor. Symptoms such as painful urination were recognized by 390 participants, which is a significant proportion. When experiencing UTI symptoms, 285 (66.1%) indicated they would go to the hospital. Significant associations were found between awareness, knowledge, and socio-demographic factors.

Conclusion

This study highlights the need for increased awareness and knowledge about UTIs among Saudi Arabian citizens in Alhassa. Tailored educational interventions are essential to correct misconceptions, promote accurate risk factor awareness, and encourage appropriate management strategies. Public health campaigns can contribute to reducing the burden of UTIs in the community.

## Introduction

Urinary tract infections (UTIs) are a common health problem worldwide, affecting individuals of all ages and genders. They often result from the invasion and multiplication of pathogenic bacteria in the urinary system [1]. UTIs can lead to significant morbidity and healthcare burden and, if left untreated, may progress to severe complications such as pyelonephritis and septicemia [2]. In the United States, approximately 25% to 40% of women in the age group 20-40 have had a UTI. UTIs account for over six million patient visits to physicians annually in the United States [3]. UTIs are a significant burden on the healthcare system in the Kingdom of Saudi Arabia (KSA). They account for 10% of all infections in the country and are the second most common reason for ED admissions [4-5]. A study conducted in Malaysia revealed a moderate level of awareness and knowledge about UTIs among its participants [6]. Similarly, another study in Saudi Arabia focusing on parents also reported a moderate level of UTI knowledge [7]. Additionally, a study among pregnant women in Saudi Arabia found that 66% were aware and knowledgeable about UTIs [8]. Assessing the knowledge, awareness, and attitudes toward UTIs in the general population is crucial for developing effective prevention strategies and ensuring early detection and prompt treatment. In our study, we aim to investigate these factors regarding UTIs among Saudi Arabian citizens residing in Al-Ahsa.

## Materials and methods

Study design

A descriptive cross-sectional study was conducted to assess the level of knowledge, awareness, and attitudes toward UTIs among Saudi Arabian citizens residing in Al-Ahsa, a city located in the Eastern Province of Saudi Arabia.

Inclusion and exclusion criteria

Inclusion criteria were as follows: Saudi Arabian citizens residing in Alhassa, a city in the Eastern Province of Saudi Arabia, aged 18 years or older, and willing to provide informed consent. Exclusion criteria included individuals who were unable or unwilling to provide informed consent, non-Saudi Arabian citizens or those not residing in Al-Ahsa, and individuals with cognitive impairments or language barriers that could hinder their ability to accurately complete the surveys or questionnaires.

Data collection methods and tools

The data for this study was collected through questionnaires administered to the participants. The questionnaires were designed specifically for this study to assess the level of knowledge, awareness, and attitudes toward UTIs among the target population. The questions were developed based on current literature, guidelines, and expert opinions in the field. The questionnaires were distributed in electronic formats via social media platforms. The questionnaire was divided into three sections: The first section included informed consent, followed by the second section, which included questions regarding socio-demographic factors such as age, gender, education level, occupation, and income. The third section asked about knowledge, awareness, and attitudes regarding UTIs.

Data analysis

The data collected from the questionnaires were analyzed and stored using IBM® SPSS® Statistics, a powerful statistical software platform.

Research ethics approval

The research proposal was submitted to the King Faisal University committee for approval with code number KFU-REC-2022-SEP-ETHICS1,322. The study was conducted in accordance with applicable national and international ethical guidelines and regulations. It was essential to uphold these ethical considerations to ensure the well-being, autonomy, and confidentiality of the study participants, as well as the integrity and credibility of the research findings.

## Results

The study involved 445 participants. Among them, the majority, 279 (62.7%), were male, while 166 (37.3%) were female. In terms of marital status, 226 (50.8%) were married, 208 (46.7%) were single, 6 (1.3%) were divorced, and 5 (1.1%) were widowed. Concerning educational attainment, 195 (43.8%) held university degrees, 100 (22.5%) completed high school, 128 (28.8%) had postgraduate degrees, 12 (2.7%) had intermediate education, and 10 (2.2%) had primary education. With regards to residence, 378 (84.9%) lived in urban areas, while 67 (15.1%) resided in rural villages. In the context of employment, 375 (84.3%) worked in non-healthcare occupations, and 70 (15.7%) were in healthcare-related fields. Regarding monthly income, approximately 241 (54.2%) reported earnings below 5000 SR, 100 (22.5%) earned between 5000 and 10000 SR, 56 (12.6%) earned between 10001 and 15000 SR, and 48 (10.8%) earned more than 15000 SR (Table 1).

**Table 1:** Sociodemographic data.

Variables	Categories	N	%
Gender	Male	279	62.7
	Female	166	37.3
Marital status	Single	208	46.7
	Married	226	50.8
	Divorced	6	1.3
	Widowed	5	1.1
Education level	Primary	10	2.2
	Intermediate	12	2.7
	High school	100	22.5
	University	195	43.8
	Postgraduate	128	28.8
Residence	City	378	84.9
	Village	67	15.1
Career	Health care worker	70	15.7
	Non-Health care worker	375	84.3
Monthly income	Less than 5000 SR	241	54.2
	From 5000 to 10000 SR	100	22.5
	From 10001 to 15000 SR	56	12.6
	More than 15000 SR	48	10.8

The majority of participants, 302 (70.1%), demonstrated knowledge about urinary tract infections (UTIs), while 133 (29.9%) lacked awareness. Additionally, 221 (49.7%) defined a UTI as inflammation of both the urethra and bladder, 74 (16.6%) believed it involved only the urethra, 11 (2.5%) thought it was solely the bladder, and 139 (31.2%) did not identify it as any of the above. Regarding the most common cause of UTIs, the majority, 261 (58.7%), attributed it to bacteria, followed by 69 (15.5%) who cited hygiene, 53 (11.9%) fungi, 44 (9.9%) viruses, and 18 (4%) protozoa. Common UTI symptoms reported included painful urination by 390 participants (87.6%), urgency by 272 (61.1%), red urine by 214 (48%), abdominal pain by 150 (33.7%), frequent urination by 141 (31.7%), back pain by 129 (28.9%), fever by 85 (19.1%), and constipation by 40 (9%). Additionally, 26 (5.8%) were uncertain about the symptoms. Factors increasing UTI risk were identified as delaying urination by 334 (75%), drinking minimal water by 279 (62.7%), inadequate perineum cleaning from front to back by 216 (48.5%), urinating after eating by 22 (4.9%), drinking substantial water by 17 (3.8%), and 41 (9.2%) remained uncertain (Table 2).

**Table 2:** The knowledge about the urinary tract infection.

Knowledge item	No	%
Do you know what a urinary tract infection (UTI) is?		
Yes	302	70.1
No	133	29.9
What is a urinary tract infection (UTI)?		
Inflammation of the urethra	74	16.6
Inflammation of the bladder	11	2.5
Both of the above	221	49.7
Neither of the above	139	31.2
What is the most common cause of urinary tract infection (UTI)?		
Bacteria	261	58.7
Virus	44	9.9
Fungi	53	11.9
Protozoa	18	4
Hygiene	69	15.5
Which symptoms occur with urinary tract infection (UTI)?		
Pain in urination	390	27.0
Red urine	214	14.8
Abdominal pain	150	10.4
Fever	85	5.8
Back pain	129	8.9
Frequent urination	141	9.7
Urgency	272	18.8
Constipation	40	2.8
Don't know	26	1.8
Which factors increase the chances of having a urinary tract infection (UTI)?		
Not cleaning the perineum properly	216	23.8
Urinating after eating	22	2.4
Drinking a large amount of water	17	1.9
Drinking a small amount of water	279	30.7
Delaying urination	334	36.7
Don't know	41	4.5

Results revealed that 285 participants (64%) believed going to the hospital was the best action for dealing with UTIs, while 118 (26.5%) thought drinking more water would help. A smaller percentage advocated for direct measures like taking antibiotics (15 participants, 3.4%), taking more showers (2 participants, 0.4%), resting at home (1 participant, 0.2%), and taking analgesics (1 participant, 0.2%), with 23 participants (5.2%) being unsure. Approximately 301 participants (70%) considered UTIs common, whereas 19 (4.4%) disagreed, and 110 (25.6%) were unsure. Furthermore, 200 (50.5%) believed UTIs affect females more than males, 57 (14.4%) thought they affect males more, 62 (15.7%) believed the impact was equal for both genders, and 77 (19.4%) were unsure. Regarding the seriousness of UTIs, 321 participants (76.4%) regarded them as serious, while 36 (8.6%) did not, and 63 (15%) were unsure. Expected complications identified included recurrent UTIs (185 participants, 41.6%) and impact on quality of life (166 participants, 37.3%) (Table 3).

**Table 3:** Perception and attitude about urinary tract infection.

Attitude items	N	%
What do you think is the best way to deal with a urinary tract infection (UTI)?		
Go to the hospital	285	64
Take rest at home	1	0.2
Take antibiotics directly	15	3.4
Take an analgesic	1	0.2
Drink more water	118	26.5
Take more showers	2	0.4
Don’t know	23	5.2
Do you think urinary tract infection (UTI) is common?		
Yes	301	70
No	19	4.4
I don't know	110	25.6
Who do you think is more affected by urinary tract infection (UTI)?		
Affects females more than males	200	50.5
Affects males more than females	57	14.4
Affects both equally	62	15.7
I don’t know	77	19.4
Do you think urinary tract infection (UTI) is serious?		
Yes	321	76.4
No	36	8.6
I don't know	63	15
What complications do you expect from a urinary tract infection (UTI)?		
Recurrent urinary tract infection (UTI)	185	41.6
Affects concurrent pregnancy	37	8.3
Affects the quality of life	166	37.3
Death	7	1.6
Weight decrease	2	0.4
Generalized edema	48	10.8

Findings indicated that 187 participants (42%) had experienced a UTI. Among them, 89 (47.6%) reported having UTIs once per year, 50 (26.7%) twice yearly, 16 (8.6%) three times yearly, and 31 (17.1%) more than three times yearly. Among those who experienced UTIs, 83 (45.5%) reported painful urination, 67 (35.8%) had a fever, 18 (9.6%) experienced abdominal pain, and 17 (9.1%) reported frequent urination. In terms of daily water intake, most participants, 210 (42.7%), consumed approximately 3 to 4 bottles of water per day, 132 (29.7%) drank 1 to 2 bottles, 72 (16.2%) consumed 5 to 6 bottles, and 51 (11.5%) drank more than 6 bottles per day. Roughly half of the participants, 235 (52.8%), reported consuming bladder-irritating fluids such as coffee and tea. Regarding actions taken when experiencing UTI symptoms, the majority, 294 (66.1%), indicated that they would go to the hospital, 113 (25.4%) said they would drink more water, 15 (3.4%) would take antibiotics directly, 8 (1.8%) would take analgesics, 3 (0.7%) would increase water intake, and only 12 (2.7%) would do nothing (Table 4).

**Table 4:** Practices and behaviors related to urinary tract infections.

Behavior items	N	%
Have you ever experienced a urinary tract infection (UTI)?		
Yes	187	42
No	258	58
If yes, how many times have you had it?		
1 time per year	89	47.6
2 times per year	50	26.7
3 times per year	16	8.6
More than 3 times per year	32	17.1
What symptoms did you notice?		
Pain in urination	85	45.5
Abdominal pain	18	9.6
Fever	67	35.8
Frequent urination	17	9.1
How many times do you drink water per day?		
1-2 bottles	132	29.7
3-4 bottles	190	42.7
5-6 bottles	72	16.2
More than 6 bottles	51	11.5
Do you drink fluids that irritate the bladder like coffee and tea?		
Yes	235	52.8
No	210	47.2
If you feel symptoms of a urinary tract infection (UTI), what do you do?		
Go to the hospital	294	66.1
Take antibiotics directly	15	3.4
Take an analgesic	8	1.8
Drink more water	113	25.4
Take more showers	3	0.7
Do nothing	12	2.7

Results showed that a higher percentage of males, 206 (74%), were aware of UTIs compared to females, 106 (64%), and this difference was statistically significant (p-value = 0.026). Among healthcare workers, 63 (90%) were knowledgeable about UTIs, which was significantly higher than the awareness level among non-healthcare workers, 249 (66%), with a p-value of less than 0.001. However, no significant associations were observed between UTI awareness and marital status, education level, residence, or monthly income (Table 5).

**Table 5:** Distribution of individuals who know what a urinary tract infection (UTI) is according to their demographic data.

Variables	Categories	Yes		No		Chi square	P-value
		N	%	N	%		
Gender	Male	206	74%	73	26%	4.947	0.026
	Female	106	64%	60	36%		
Marital status	Single	146	70%	62	30%	3.295	0.348
	Married	158	70%	68	30%		
	Divorced	3	50%	3	50%		
	Widowed	5	100%	0	0%		
Educational level	Primary	6	60%	4	40%	1.552	0.817
	Intermediate	10	83%	2	17%		
	High school	69	69%	31	31%		
	University	137	70%	58	30%		
	Post graduate	90	70%	38	30%		
Residence	City	266	70%	112	30%	0.080	0.778
	Village	46	69%	21	31%		
Career	Health care worker	63	90%	7	10%	15.679	<0.001
	Non-Health care worker	249	66%	126	34%		
Monthly income	Less than 5000 SR	169	70%	72	30%	3.306	0.347
	From 5000 to 10000 SR	73	73%	27	27%		
	From 10001 to 15000 SR	34	61%	22	39%		
	More than 15000 SR	36	75%	12	25%		

Findings revealed that among the participants, 8 (80%) with primary education, 11 (100%) with intermediate education, 79 (89%) with high school education, 133 (70%) with university education, and 90 (75%) with postgraduate education believed that UTIs are a serious condition. This association was found to be statistically significant, with a p-value of less than 0.001. However, no significant associations were observed between the perception of UTIs as a serious condition and factors such as gender, marital status, residence, occupation, or monthly income (Table 6).

**Table 6:** Distribution of individuals who believe a urinary tract infection (UTI) is serious according to their demographic data.

Variables	Categories	Yes		No		I don't know		Chi square	P-value
		N	%	N	%	N	%		
Gender	Male	200	75%	29	11%	39	15%	4.785	0.091
	Female	121	80%	7	5%	24	16%		
Marital status	Single	141	72%	19	10%	36	18%	6.244	0.396
	Married	173	81%	16	7%	25	12%		
	Divorced	3	60%	1	20%	1	20%		
	Widowed	4	80%	0	0%	1	20%		
Educational level	Primary	8	80%	1	10%	1	10%	25.774	< 0.001
	Intermediate	11	100%	0	0%	0	0%		
	High school	79	89%	0	0%	10	11%		
	University	133	70%	28	15%	29	15%		
	Post graduate	90	75%	7	6%	23	19%		
Residence	City	271	75%	31	9%	59	16%	3.714	0.156
	Village	50	85%	5	8%	4	7%		
Career	Health care worker	51	76%	3	4%	13	19%	2.590	0.274
	Non-Health care worker	270	76%	33	9%	50	14%		
Monthly income	Less than 5000 SR	165	72%	24	10%	40	17%	7.705	0.261
	From 5000 to 10000 SR	76	81%	8	9%	10	11%		
	From 10001 to 15000 SR	43	83%	1	2%	8	15%		
	More than 15000 SR	37	82%	3	7%	5	11%		

Results indicated that the percentage of males, 131 (47%), who had experienced UTIs was higher than that of females, 56 (34%), and this difference was statistically significant (p-value = 0.006). The highest percentage of individuals reporting UTI experiences were widowed, 3 (60%), followed by married, 123 (54%), divorced, 3 (50%), and single individuals, 58 (28%). This association also showed statistical significance (p-value < 0.001). In terms of education levels, the highest percentage of individuals who had experienced a UTI had intermediate education, 10 (83%), followed by those with primary and high school education, 50 (50%), university education, 77 (39%), and postgraduate education, 45 (35%). This association was statistically significant, with a p-value of 0.006. Non-healthcare workers, 167 (45%), were more likely to have experienced a UTI compared to healthcare workers, 20 (29%), and this difference was statistically significant (p-value = 0.013). However, no significant association was found between individuals who had ever experienced a UTI and their residence or monthly income (Table 7).

**Table 7:** Distribution of participants who have experienced a urinary tract infection (UTI) according to their demographic data.

Variables	Categories	Yes		No		Chi square	P-value
		N	%	N	%		
Gender	Male	131	47%	148	53%	7.464	0.006
	Female	56	34%	110	66%		
Marital status	Single	58	28%	150	72%	32.153	< 0.001
	Married	123	54%	103	46%		
	Divorced	3	50%	3	50%		
	Widowed	3	60%	2	40%		
Educational level	Primary	5	50%	5	50%	14.270	0.006
	Intermediate	10	83%	2	17%		
	High school	50	50%	50	50%		
	University	77	39%	118	61%		
	Post graduate	45	35%	83	65%		
Residence	City	157	42%	221	58%	0.246	0.620
	Village	30	45%	37	55%		
Career	Health care worker	20	29%	50	71%	6.169	0.013
	Non-Health care worker	167	45%	208	55%		
Monthly income	Less than 5000 SR	95	39%	146	61%	3.628	0.30
	From 5000 to 10000 SR	45	45%	55	55%		
	From 10001 to 15000 SR	29	52%	27	48%		
	More than 15000 SR	18	38%	30	63%		

## Discussion

UTIs are a significant global health concern [1]. Our study aimed to assess the awareness, knowledge, and attitudes regarding UTIs among Saudi Arabian citizens residing in Al-Ahsa. The results reveal several significant findings and implications. Our study found that most participants displayed a reasonable level of knowledge regarding UTIs. Specifically, more than two-thirds of participants correctly identified what a UTI is, and when defining UTIs, half of them associated them with inflammation of both the urethra and bladder. Almost two-thirds of participants recognized bacteria as the leading cause of UTIs. Common UTI symptoms reported included "painful urination," "urgency," and "red urine." Factors contributing to increased UTI risk included behaviors like "delaying urination," "drinking a small amount of water," and "neglecting perineum hygiene." Additionally, almost two-thirds believed that seeking hospital care was the best response to UTIs, contrasted with prior research. Furthermore, a notable proportion considered UTIs to be common, and most participants acknowledged the seriousness of UTIs. The most expected complications were "recurrent UTIs" and "affecting the quality of life." We found that most participants demonstrated a reasonable level of knowledge regarding UTIs. Specifically, more than two-thirds correctly identified what a UTI is, aligning with previous studies conducted by Al-Mutairi NZ and Amara FM in 2015 in the Hail region, Saudi Arabia, and Badran YA et al. in 2016 among Saudi women [9,10]. When defining UTIs, almost half of the participants associated UTIs with inflammation of both the urethra and bladder. This differs from the findings of Badran YA et al. [10], where only around a third of participants provided the same response. These variations may be attributed to differences in cultural and educational backgrounds, as well as sample size and demographics. In terms of the most common cause of UTIs, almost two-thirds of participants identified bacteria, consistent with Al-Mutairi NZ and Amara FM's findings in 2015, suggesting a general understanding of UTI etiology among the population [9]. The responses differed slightly from previous studies when asked about common UTI symptoms. In our study, painful urination, urgency, and red urine were reported as the most common symptoms. However, Badran YA et al. found that frequent urge to urinate and burning sensation were the most frequently reported symptoms [10]. These differences might be influenced by cultural variations, healthcare access, and perception of symptom severity.

The study identified various factors contributing to an increased risk of UTIs, including delaying urination, drinking a small amount of water, and neglecting to clean the perineum thoroughly from front to back. These findings are congruent with the results of Al-Mutairi NZ and Amara FM, highlighting the importance of personal hygiene and hydration in preventing UTIs [9]. In terms of managing UTIs, almost two-thirds of our participants believed that going to the hospital was the best course of action. This contrasts with the findings of Badran YA et al., where nearly half of the participants held the same belief [10]. These disparities could be attributed to differences in healthcare systems, access to medical services, and cultural beliefs about seeking treatment. A high percentage of our participants considered UTIs to be common, a finding that is consistent with the research of Al-Mutairi et al. in 2015 [9]. This perception reflects the prevalence of UTIs in the general population and underscores the importance of awareness and preventive measures. While most participants recognized the seriousness of UTIs, the anticipated complications varied, with recurrent UTIs and impact on quality of life being the most expected. These findings are in line with those reported by Badran YA et al. in 2016 [10].

Limitations

Acknowledging the limitations of our study, we note that our findings are based on self-reported data, which may be subject to recall and response bias. Additionally, the study's cross-sectional design limits our ability to establish causality.

Recommendations

To address these limitations, we recommend further research using longitudinal designs to explore the effectiveness of educational interventions in improving UTI awareness and knowledge among the Saudi Arabian population. Moreover, healthcare authorities should consider developing and implementing evidence-based public health campaigns targeting misconceptions and promoting accurate UTI awareness.

## Conclusions

In conclusion, our study underscores the necessity of enhancing awareness and knowledge about UTIs among the citizens of Al-Ahsa, Saudi Arabia. Addressing misconceptions, emphasizing accurate risk factors and symptoms, and promoting appropriate management strategies are essential for reducing the burden of UTIs in the community. Developing public health campaigns and targeted educational interventions could significantly improve awareness about UTIs, ultimately leading to improved healthcare outcomes.

## References

[1] Fettig J, Swaminathan M, Murrill CS, Kaplan JE (2014). Global epidemiology of HIV. Infect Dis Clin North Am.

[2] Sabih A, Leslie SW (2023). Complicated urinary tract infections. https://www.ncbi.nlm.nih.gov/books/NBK436013/.

[3] Hsiao CJ, Cherry DK, Beatty PC (2010). National Ambulatory Medical Care Survey: 2007 summary. Natl Health Stat Report.

[4] Alanazi MQ, Al-Jeraisy MI, Salam M (2015). Prevalence and predictors of antibiotic prescription errors in an emergency department, Central Saudi Arabia. Drug Healthc Patient Saf.

[5] Alrashid S, Ashoor R, Alruhaimi S, Hamed A, Alzahrani S, Al Sayyari A (2022). Urinary tract infection as the diagnosis for admission through the emergency department: its prevalence, seasonality, diagnostic methods, and diagnostic decisions. Cureus.

[6] Neni WS, Subrain G, Shamshir Khan MS (2019). Awareness, knowledge, and attitude of Government Secondary School students on the use of antibiotics in Shah Alam, Malaysia. J Pub Health.

[7] Almatrafi MA, Sindi L, Alshehri M (2022). Parental knowledge and awareness of childhood urinary tract infections: a cross sectional survey. Patient Prefer Adherence.

[8] Asiri A, Alasiri A, Asiri M (2022). Awareness of Saudi women about causes of urinary tract infection and its complications in pregnant women in Asir Region. King Khalid Univ J Health Sci.

[9] Al-Mutairi NZ, Amara FM (2015). Assessment of public knowledge, attitude, and practice toward urinary tract infections in the Hail Region, Saudi Arabia. Int J Med Sci Public Health.

[10] Badran YA, Kamel NA, Mostafa NA (2016). Urinary tract infection knowledge, attitude, and practice among Saudi women. Saudi Med J.

